# Lung function in adults and future burden of obstructive lung diseases in a long-term follow-up

**DOI:** 10.1038/s41533-020-0169-z

**Published:** 2020-03-26

**Authors:** Lene Maria Ørts, Bodil Hammer Bech, Torsten Lauritzen, Anders Helles Carlsen, Annelli Sandbæk, Anders Løkke

**Affiliations:** 1 Section for General Practice, Department of Public Health, Aarhus University, Aarhus, Denmark; 2 Research Unit for Epidemiology, Department of Public Health, Aarhus University, Aarhus, Denmark; 3 Research Unit for General Practice, Aarhus University, Aarhus, Denmark; 4 Department of Medicine, Hospital Little Belt, Vejle, Denmark

**Keywords:** Preventive medicine, Respiratory signs and symptoms, Chronic obstructive pulmonary disease, Health occupations, Epidemiology

## Abstract

Spirometry is recommended in symptomatic smokers to identify obstructive lung diseases. However, it is unknown whether there are certain characteristics that can be used to identify the individual risk of developing obstructive lung diseases. The aim of this study was to examine the association between lung function in adults and burden of lung diseases throughout 27 years of follow-up. We performed a cohort study among individuals aged 30–49 years at baseline (1991). Spirometry measurements were divided into three groups: (1) FEV_1_/FVC < 70, (2) FEV_1_/FVC: 70–75, (3) FEV_1_/FVC > 75 (reference). Using negative binominal regression, the burden of lung diseases was measured by contacts to general practice, hospitalisations, redeemed respiratory medicine and socioeconomic parameters between 1991 and 2017. A total of 905 citizens were included; mean age of 40.3 years, 47.5% were males and 51.2% were smokers at baseline. The group with an FEV_1_/FVC: 70–75 received more respiratory medicine (IRR = 3.37 (95% CI: 2.69–4.23)), had lower income (IRR = 0.96 (95% CI: 0.93–0.98)), and had more contacts to general practice (IRR = 1.14 (95% CI: 1.07–1.21)) and hospitals for lung diseases (IRR = 2.39 (95% CI: 1.96–5.85)) compared to the reference group. We found an association between lung function and the future burden of lung diseases throughout 27 years of follow-up. In particular, adults with an FEV_1_/FVC: 70–75 need extra attention in the case finding.

## Introduction

Obstructive lung diseases (OLDs) are common and serious diseases, representing a growing public health challenge worldwide^1^. The prevalence of asthma varies between 15% and 18% depending on the way of diagnosing^2^. Chronic obstructive pulmonary disease (COPD) is estimated to become the third most prevalent cause of death worldwide in 2030, but longitudinal studies of the socioeconomic consequences of individuals at risk of developing COPD are sparse^3–5^. This is remarkable, as COPD is known to be a substantial burden for healthcare system, the patients and their relatives^3–6^.

Different approaches to improve case finding in OLD have been investigated^7–10^. Especially among younger individuals, it can be difficult to predict the future risk of developing OLD, as disease progression is usually slow and can be influenced by more risk factors. Furthermore, there are no available studies that follow younger individuals in a longitudinal perspective with respect to both clinical, healthcare and sociodemographic parameters.

Underdiagnosing is a substantial problem among adults with OLD^11^. In Denmark, as in most other European countries, the general practitioner (GP) is in the frontline to perform spirometry in case finding. The Global Initiative for Chronic Obstructive Lung Disease (GOLD) strategy recommends use of initial spirometry for early detection in symptomatic smokers and individuals with a predisposition to lung diseases^12^. The US preventive Service Task Force does not recommend screening for COPD in asymptomatic smokers, mainly because smoking cessation rates did not seem to improve by screening^7,13^. Nevertheless, individuals with undiagnosed COPD have an increased risk of pneumonia, exacerbations and death, suggesting that studies regarding case finding are needed^6^.

Spirometry measurement is the gold standard to diagnose OLD, and according to the GOLD strategy document^12^, the definition of abnormal lung function is a forced expiratory volume in 1 s (FEV_1_)/a forced vital capacity (FVC) < 70. Physiologically, younger individuals have a high ratio and must decrease a disproportionate amount before dropping below FEV_1_/FVC < 70^14,15^.

We hypothesised that individuals with an FEV_1_/FVC < 70 at baseline had a higher burden of OLD and a poorer sociodemographic profile during the follow-up period compared to the reference group. Furthermore, because of their young age at baseline, we hypothesised that individuals with an FEV_1_/FVC: 70–75 were more likely to behave as the group with FEV_1_/FVC < 70, than the reference group in a long-term follow-up.

Therefore, the aim of this study was to examine the association between lung function in individuals aged 30–49 years at baseline and the burden of OLD, both from a physiological and socioeconomical perspective, throughout 27 years of follow-up. The burden of OLD was based on the number of consultations in general practice, lung-related contacts to the hospital and redeemed prescriptions for respiratory medication in the period from 1991 until 2017. Furthermore, we determined the sociodemographic factors based on the level of income, employment and education.

## Results

### Baseline characteristics

We followed 905 citizens for 27 years (23,538 person years). At baseline, the group with an FEV_1_/FVC < 70 and the group with an FEV_1_/FVC: 70–75 were slightly more likely to be male and smokers as well as to report more airway symptoms than the reference group (FEV_1_/FVC > 75) (Table 1). There were more individuals with a low level of education and income level in the group with an FEV_1_/FVC < 70 (Table 1).

**Table 1:** Baseline characteristics among 905 participants included in 1991.

	FEV_1_/FVC	FEV_1_/FVC	FEV_1_/FVC	Total	Missing
	<70	70–75	>75		*n*/*N*
*N* (%)	52 (5.7)	95 (10.5)	758 (83.8)	905 (100.0)	0/905
Sex (male), *n* (%)	30 (57.7)	54 (56.8)	346 (45.6)	430 (47.5)	0/905
Age, mean (SD)	41.8 (6.0)	41.0 (5.8)	40.1 (5.6)	40.3 (5.7)	0/905
Lung function					
FEV_1_/FVC, mean (SD)	65.1 (4.5)	72.8 (1.4)	82.0 (4.3)	80.1 (6.2)	0/905
FEV_1_ % predicted, mean (SD)	80.0 (12.9)	92.6 (10.1)	100.2 (11.0)	98.2 (12.2)	0/905
Airway symptoms within a year, *n* (%)					
No symptoms, *n* (%)	24 (46.2)	59 (62.1)	576 (76.0)	659 (72.8)	
Light symptoms, *n* (%)	20 (38.5)	30 (31.6)	156 (20.6)	206 (22.8)	
Severe symptoms, *n* (%)	8 (15.4)	6 (6.3)	26 (3.4)	40 (4.4)	0/905
Smoking status, *n* (%)					
Never smoker	8 (15.4)	23 (24.5)	295 (39.1)	326 (36.2)	
Current smoker	39 (75.0)	57 (60.6)	365 (48.4)	461 (51.2)	
Former smoker	5 (9.6)	14 (14.9)	94 (12.5)	113 (12.6)	5/905
Education (years), *n* (%)					
0–10 (low)	19 (37.3)	29 (31.9)	201 (27.1)	249 (28.2)	
10–15 (medium)	22 (43.1)	43 (47.3)	370 (49.9)	435 (49.3)	
>15 (high)	10 (19.6)	19 (20.9)	170 (22.9)	199 (22.5)	22/905
Income, 1000 euro, *n* (%)					
Low tertile (0–12)	24 (46.2)	31 (32.6)	261 (34.4)	316 (34.9)	
Middle tertile (13–16)	12 (23.1)	33 (34.7)	265 (35.0)	310 (34.3)	
High tertile (>17)	16 (30.8)	31 (32.6)	232 (30.6)	279 (30.8)	0/905

Data are *n* (%) or mean (SD).

*FEV*_*1*_ forced expiratory volume in 1 s, *FVC* forced vital capacity, *SD* standard deviation.

### Contacts to GPs

**Fig. 1:**
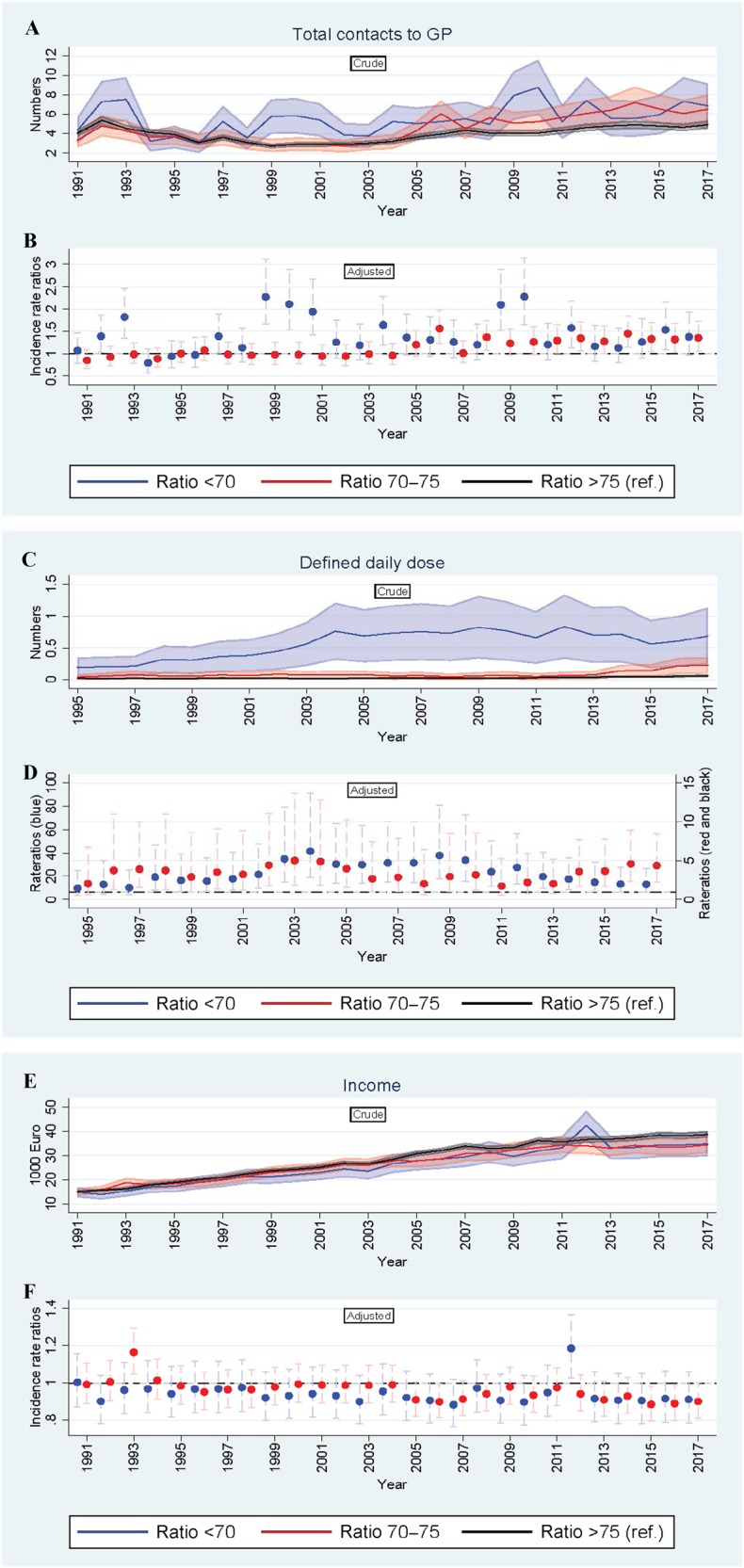
Mean numbers and Incidence rate ratios 1991–2017. **a**–**f** The figure consists of six graphs showing the descriptive correlation between lung function at baseline (exposure) and development of medical health condition on different parameters. Outcomes in 1-year intervals are: number of contacts to general practitioner from 1991 to 2017 (panel **a**, **b**), redeemed prescriptions for respiratory medicine from 1995 to 2017 (panel **c**, **d**) and income level from 1991 to 2017 (panel **e**, **f**). *X*-axis: time (years), *Y*-axis: outcome (numbers). Unadjusted, crude mean numbers are shown in the top panel and adjusted IRRs (95% CI) in the bottom panels. Group three (FEV_1_/FVC>75) is the reference group and the regressions is adjusted for sex, age and smoking status. GP general practitioner, FEV_1_ forced expiratory volume in 1s, FVC forced vital capacity.

**Fig. 2:**
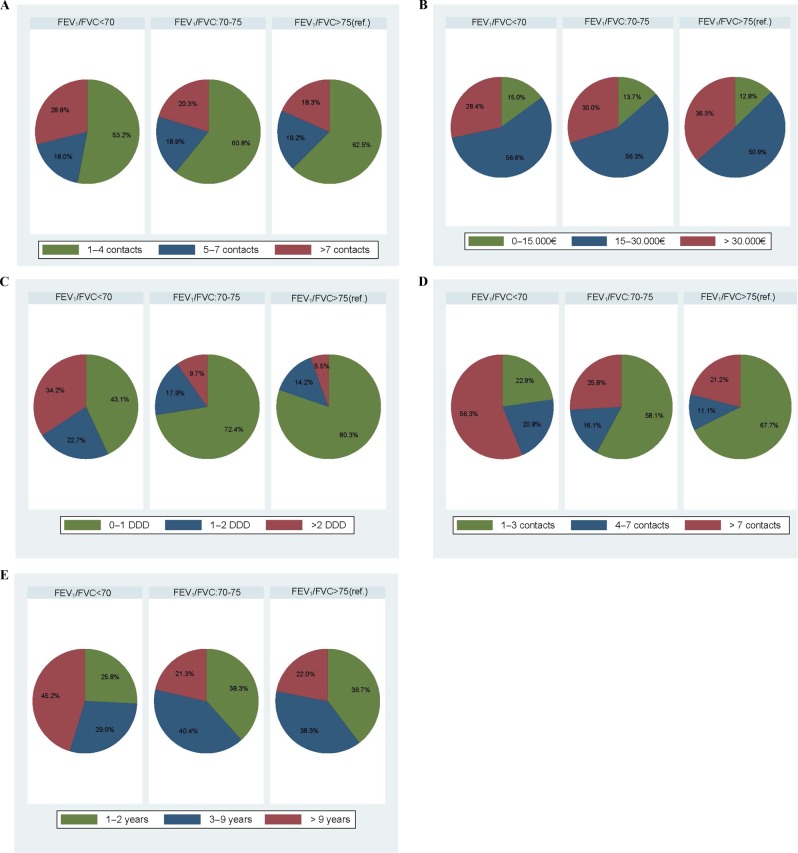
Distribution of contacts to general practice, level of income, redeemed prescriptoons of respiratory medicine, lung-related hospital contacts and unemployement registrations. **a**–**e** The distribution across the three groups ((1) FEV_1_/FVC<70, (2) FEV_1_/FVC: 70–75 and (3) FEV_1_/FVC>75) is shown in percentages. Panel **a** shows the annual number of contacts to general practice categorised into 3 groups: 1–4 contacts, 5–7 contacts, and >7 contacts. Panel **b** shows the annual level of income categorised into 3 groups; 0–15,000€, 15–30,000€, and >30,000€. Panel **c** shows the redeemed prescriptions of respiratory medicine categorised into 3 groups; 0–1 DDD, 1–2 DDD, and >2 DDD. Panel **d** shows the amount of lung-related hospital contacts categorised into 3 groups; 1–3 contacts, 4–7 contacts, and >7 contacts. Panel **e** shows the number of years being unemployed categorised into 3 groups; 1–2 years, 3–9 years, and >9 years. FEV_1_ forced expiratory volume in 1s, FVC forced vital capacity, DDD defined daily doses, GP general practitioner.

The total number of GP contacts during the follow-up period was 99,126. The average unadjusted number of GP contacts for each individual was 4.0 contacts per year. On average, individuals with an FEV_1_/FVC < 70 had 40% more contacts during the period (adjusted incidence rate ratio (aIRR) = 1.40, 95% confidence interval (CI) 1.28; 1.54) and individuals with an FEV_1_/FVC: 70–75 had 14% more contacts per year (aIRR = 1.14, 95% CI 1.07; 1.21) than the reference group (Table 2). For individuals with an FEV_1_/FVC: 70–75, we only found a statistically significant association at the end of the study period between 2004 and 2017 (aIRR = 1.24 (95% CI 1.14; 1.35) where individuals were aged 43–63 years (Table 2, Figs 1a, b and 2a).

**Table 2:** Crude and adjusted incidence rate ratios (1991–2017).

Lung function	Years	Crude IRR (95% CI)	Adjusted IRR^a^ (95% CI)
GP contacts			
FEV_1_/FVC (<70)	1991–2017	1.39 (1.28; 1.52)	1.40 (1.28; 1.54)
	1991–2003	1.36 (1.17; 1.57)	1.40 (1.23; 1.68)
	2004–2017	1.44 (1.30; 1.59)	1.41 (1.26; 1.58)
FEV_1_/FVC (70–75)	1991–2017	1.14 (1.07; 1.21)	1.14 (1.07; 1.21)
	1991–2003	0.95 (0.88; 1.03)	0.99 (0.92; 1.07)
	2004–2017	1.29 (1.18; 1.41)	1.24 (1.14; 1.35)
Income, yearly			
FEV_1_/FVC (<70)	1991–2017	0.90 (0.87; 0.93)	0.93 (0.91; 0.97)^b^
	1991–2003	0.92 (0.89; 0.95)	0.94 (0.91; 0.97)^b^
	2004–2017	0.90 (0.86; 0.95)	0.94 (0.90; 0.98)^b^
FEV_1_/FVC (70–75)	1991–2017	0.93 (0.91; 0.96)	0.96 (0.93; 0.98)^b^
	1991–2003	0.98 (0.95; 1.01)	0.99 (0.96; 1.02)^b^
	2004–2017	0.91 (0.88; 0.94)	0.93 (0.91; 0.96)^b^
Daily defined dose			
FEV_1_/FVC (<70)	1995–2017	20.44 (16.88; 24.76)	21.12 (17.38; 25.66)
	1995–2003	13.70 (8.82; 21.30)	17.38 (10.94; 27.62)
	2004–2017	23.65 (19.03; 29.39)	24.02 (19.34; 29.84)
FEV_1_/FVC (70–75)	1995–2017	3.01 (2.40; 3.77)	3.37 (2.69; 4.23)
	1995–2003	2.86 (1.78; 4.59)	3.58 (2.25; 5.69)
	2004–2017	2.91 (2.22; 3.81)	3.21 (2.45; 4.21)
Hospital contacts			
FEV_1_/FVC (<70)	1991–2017	19.37 (11.66; 32.17)	14.30 (8.43; 24.25)
	1991–2003	10.56 (4.25; 26.25)	3.54 (1.32; 9.49)
	2004–2017	26.10 (13.40; 50.82)	18.67 (10.27; 33.97)
FEV_1_/FVC (70–75)	1991–2017	2.61 (1.47; 4.62)	3.39 (1.96; 5.85)
	1991–2003	0.95 (0.32; 2.89)	1.73 (0.56; 5.37)
	2004–2017	2.91 (1.38; 6.15)	3.38 (1.71; 6.69)
Unemployment			
FEV_1_/FVC (<70)	1991–2017	2.26 (2.03; 2.50)	2.04 (1.84; 2.26)
	1991–2003	2.05 (1.77; 2.37)	1.79 (1.55; 2.08)
	2004–2017	2.57 (2.22; 2.97)	2.42 (2.10; 2.78)
FEV_1_/FVC (70–75)	1991–2017	1.06 (0.94; 1.19)	1.08 (0.96; 1.21)
	1991–2003	1.07 (0.92; 1.25)	1.08 (0.92; 1.25)
	2004–2017	1.04 (0.87; 1.25)	1.06 (0.89; 1.28)

*FEV*_*1*_ forced expiratory volume in 1 s, *FVC* forced vital capacity, *GP* general practitioner, *IRR* incidence rate ratios.

^a^Adjusted for sex, age and smoking status.

^b^Adjusted for sex, age, smoking status and education level.

### Lung-related contacts to the hospital

During follow-up, there were 1979 lung-related contacts to the hospital divided between 178 individuals (Table 3). Of these, 48 individuals were from the group with an FEV_1_/FVC < 70 (92% of the individuals in the group), 31 individuals from the group with an FEV_1_/FVC: 70–75 (33% of the individuals in the group) and 99 individuals from the group with an FEV_1_/FVC > 75 (13% of the individuals in the group) (Fig. 2d, Table 3 and Supplementary Table 2). The group with an FEV_1_/FVC: 70–75 had 3.39 times more lung-related contacts to the hospital than the reference group after adjustment for covariates (aIRR = 3.39, 95% CI 1.96; 5.85; Table 2).

**Table 3:** Lung-related contacts to the hospital (1991–2017).

	FEV_1_/FVC < 70, *N* (%)	FEV_1_/FVC: 70–75, *N* (%)	FEV_1_/FVC > 75, *N* (%)
0 contact	4 (7.7)	64 (67.4)	659 (86.9)
1–3 contacts	11 (21.2)	18 (18.9)	67 (8.8)
4–7 contacts	10 (19.2)	5 (5.3)	11 (1.5)
>7 contacts	27 (51.9)	8 (8.4)	21 (2.8)

The table shows the exact number of individuals with a hospital contact during the period.

*FEV*_*1*_ forced expiratory volume in 1 s, *FVC* forced vital capacity.

### Respiratory medicine

The total number of redeemed prescriptions for respiratory medicine during follow-up was 9082 divided between 184 individuals. The average unadjusted number of daily defined doses for the whole period was 1.28 dose per day ranging between 0.01 and 11.00 among those who received respiratory medicine. The group with an FEV_1_/FVC < 70 received much more respiratory medicine than the reference group during the whole period (Table 2). The group with an FEV_1_/FVC: 70–75 received 3.37 times more respiratory medicine than the reference group after adjustments for covariates (aIRR: 3.37 (95% CI: 2.69; 4.23) (Table 2 and Supplementary Table 3).

### Income

The average annual income was 15,222 euro at baseline and 34,511 euro in 2017. The group with an FEV_1_/FVC < 70 had a 7% lower income (aIRR = 0.93, 95% CI 0.91; 0.97) than the reference group during the whole period (Table 2). The group with an FEV_1_/FVC: 70–75 had a 4% lower income after adjustment for covariates (aIRR = 0.96, 95% CI 0.93; 0.98) than the reference group (Table 2). The difference in income was most pronounced at the end of the study period from 2004 to 2017 (Fig. 1e, f).

### Unemployment registrations

The total number of unemployment registrations was 2718, and a total of 428 participants had a least one registered unemployment period during follow-up (Table 4). Of these, 31 individuals were from the group with an FEV_1_/FVC < 70 (60% of the individuals in the group); 47 individuals were from the group with an FEV_1_/FVC: 70–75 (49% of the individuals in the group) and 350 individuals from the group with an FEV_1_/FVC < 75 (46% of the individuals in the group). The distribution across the groups is shown in percentages in Fig. 2e. The group with an FEV_1_/FVC < 70 had 2.04 times more registered unemployment periods after adjustment for covariates (aIRR = 2.04, 95% CI 1.84; 2.26) than the reference group (Table 2).

**Table 4:** Individuals being unemployed (1991–2017).

	FEV_1_/FVC < 70, *N* (%)	FEV_1_/FVC: 70–75, *N* (%)	FEV_1_/FVC > 75, *N* (%)
0 year	21 (40.4)	48 (50.5)	408 (53.8)
1–2 years	8 (15.4)	18 (19.0)	139 (18.3)
3–9 years	9 (17.3)	19 (20.0)	134 (17.7)
>9 years	14 (26.9)	10 (10.5)	77 (10.2)

The table shows the exact number of individuals registered with a period of unemployment during the period.

*FEV*_*1*_ forced expiratory volume in 1 s, *FVC* forced vital capacity.

## Discussion

The main finding of this study is that individuals with an FEV_1_/FVC: 70–75 have a higher risk of developing OLD, have a lower level of income and a higher degree of unemployment in a longitudinal perspective compared with individuals with an FEV_1_/FVC > 75. The group with FEV_1_/FVC: 70–75 redeemed 3.37 times more respiratory medicine, had a 4% lower income, 14% more contacts to general practice and 239% more hospital contacts due to lung diseases compared to individuals with an FEV_1_/FVC > 75. The group with an FEV_1_/FVC < 70 was affected on all parameters as expected. When dividing the 27-year follow-up period into two, it is clear that the difference is higher as individuals grew older.

We chose to focus on the group with an FEV_1_/FVC: 70–75 for several reasons. First, the GOLD strategy^12^ defines abnormal lung function as FEV_1_/FVC < 70 and most of the COPD study thus chose this cut-off in their analyses. Nevertheless, we expect that the affected group had already caught the attention of their GP in the follow-up and received relevant treatment. Second, we studied a young population with a physiologically high lung function^14–16^. Although, the focus was on the group with FEV_1_/FVC: 70–75, we sustained to include all three groups in the representation. Thereby we had the opportunity to compare the group with FEV_1_/FVC: 70–75 with both the affected group (FEV_1_/FVC < 70) and the healthy reference group (FEV_1_/FVC > 75) in a long-term follow-up. Our results showed that the group with FEV_1_/FVC: 70–75 is more similar to the affected group than the healthy reference group on most parameters.

A variety of approaches to identify lung diseases in primary care setting had been investigated^7,8,16–19^. Clinicians favoured the fixed ratio due to simplicity and the GOLD recommendations^20^, whereas a claim for accuracy was used among some pulmonary physiologists and researchers arguing for the lower limit of normal^7,8,16–19,21,22^. As the case finding take place in primary care, we favour the fixed ratio.

In our study, contacts to GPs are considered a proxy for health status, because GPs act as gatekeepers in the Danish healthcare system. In Denmark, the GPs treat most of the patients with respiratory symptoms^23^, and only the most severe cases are admitted to or followed at a hospital^24^. Data on hospital contacts reflect the most severe cases and support the same trend (Table 2). Knowledge about the socioeconomic status is an important aspect of the overall health status to prevent social inequality. In our study, we found that the groups with an FEV_1_/FVC < 70 and an FEV_1_/FVC: 70–75 have a significantly lower income and a higher degree of unemployment (Table 2). This finding supports the well-established connection between low levels of socioeconomic status, low health literacy and a higher degree of chronic diseases^4,5,25,26^.

At baseline, 51.2% were current smokers. Smoking rates have been declining in Denmark during the 27-year of follow-up period^27^, which may affect generalisability of the study results. We also found an increase in redeemed prescriptions for respiratory medicine in the study period (Fig. 1c). The most obvious explanation is that the participants become more ill when getting older and thereby closer to the average age of being diagnosed with COPD^6^. Another explanation is the introduction of the new inhalers e.g. fixed dose combinations of inhaled corticosteroids and inhaled beta 2-agonists during the study period and amended guidelines for treatment of OLD^28^. The combination of a decline in smoking rates and optimised treatment with relevant respiratory medicine for patients with milder COPD will reduce the likelihood of the lung function becoming affected. However, we expect the impact will be similar in the exposed groups.

To our knowledge, there are no similar studies investigating the association between impaired lung function by initial spirometry measurement in adults in combination with sociodemographic profile and burden of lung diseases. A recent study from Sweden showed that low income, unemployment and being divorced were factors related to the development of COPD^5^. Our results on long-term income reflect that of other chronic diseases. Kristensen et al.^25^ found a lower income level and a higher unemployment rate for patients with psoriatic arthritis compared with the general population both in the period prior to the diagnosis and in the years following the diagnosis. Landfeldt et al.^26^ demonstrated the same trends among patients diagnosed with multiple sclerosis.

The major strengths of this study are the high validity of the Danish registries and the complete clinical measurements obtained in the study population in 1991. Data on sociodemographic, contacts to GPs and hospitals as well as redeemed prescriptions are updated continuously and are of high quality, thus minimising information bias^29,30^. The registries contain a virtually complete 27-year follow-up, and therefore selection bias is unlikely to affect our results. However, we cannot rule out misclassification of inhalation medicine exposure, as it is uncertain if all of the redeemed prescription medicine was actually taken. Data on GP contacts are considered highly accurate as remuneration of the GPs depends on accurate registration of each individual contact and medical procedure^31^.

**Fig. 3:**
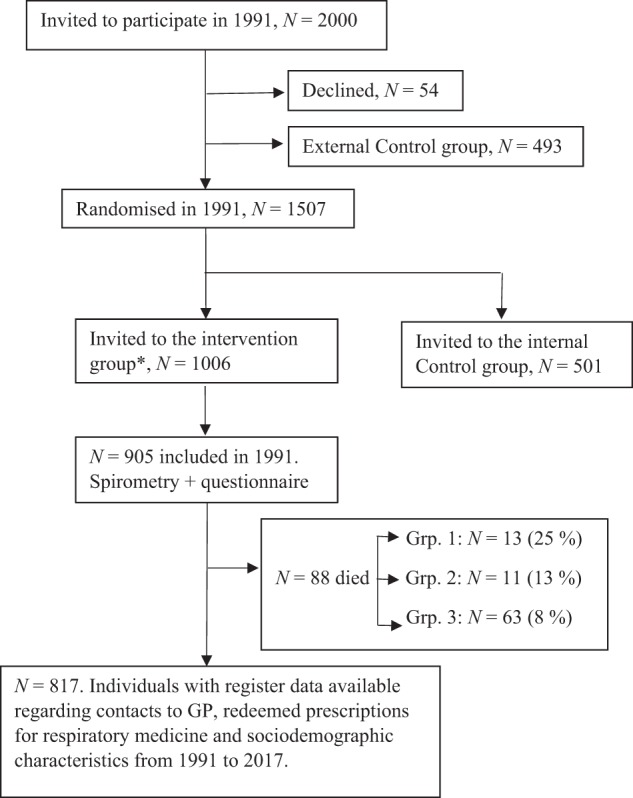
Flow diagram. GP general practitioner. Grp.1: FEV_1_/FVC < 70, Grp.2: FEV_1_/FVC: 70–75 and Grp. 3: FEV_1_/FVC > 75 (ref.).

We are aware that there are some limitations in the study. First, the long observation period may introduce selection in healthy individuals, which may have resulted in an underestimation of the number of patients with OLD. In line with this, we observed a higher mortality in the group with lower lung function and cannot thus exclude survivor bias (Fig. 3). However, as our participants were quite young at baseline, this survivor bias is likely to be smaller than that in most OLD intervention trials studying older participants^11,32^. Second, we cannot rule out registration errors in the Danish registries, although a typo would not depend on the exposure. Third, as exposure, we only had pre-bronchodilator measurements available, which may overestimate the prevalence of airflow limitation^9^. Furthermore, we do not know whether airflow limitation detected during the baseline exams was due to asthma or due to COPD. In addition, we do not know the results of spirometry tests that may have been performed during the follow-up period at the GP and during inpatient or outpatient contacts. Finally, in our statistical analyses, adjustments for potential confounders generally attenuated the IRR estimates, which may indicate confounding from these risk factors. Furthermore, confounding from unknown and unmeasured risk factors cannot be excluded.

In conclusion, this cohort study is the first to establish an association between a low FEV_1_/FVC in adults and development in the burden of lung diseases, both from a physiological and socioeconomical perspective, throughout 27 years of follow-up. The poor prognosis of individuals with an FEV_1_/FVC: 70–75 highlights the importance of implementing improved case finding initiatives to prevent development of OLD. Spirometry measurement is the preferred tool; however, more research concerning specificity and sensitivity by the use of spirometry is needed.

## Methods

### Study design and participants

We performed a cohort study based on data from the Danish Ebeltoft Health Promotion Project (EHPP)^33^ initiated in 1991. Recruitment of participants and methods are explained in detail in Supplementary Notes. In September 1991, 2000 individuals aged 30-49 years and living in the municipality of Ebeltoft were invited to attend the EHPP between January and May 1992. They were selected randomly using the civil registration number including date of birth and sex. A total of 1370 participated at baseline (participation rate 69%). Of these, 905 individuals completed the questionnaires and had a clinical examination performed.

### Procedure

In the present study, we used data on age, sex, smoking status (subdivided into never, former or current smoker) and airway symptoms (subdivided into no, light or severe symptoms) from the questionnaire. From the clinical examination, we used data on height, weight and spirometry measurement (Supplementary Notes). Informed consent was provided by every participant before entering the EHPP. The results from the questionnaire and the clinical examination were evaluated during a follow-up consultation at the GP. During the 27 years of follow-up, 88 citizens died. The deaths were censured from the analyses from the date of death. In total, 23,538 person-years were included in the analyses (Fig. 3).

The project is registered as part of the research projects, covered by the common university notification to the Danish Data Protection Agency on the processing of personal data carried out by the university, the Danish Data Protection Agency’s journal no: 2016-051-000001, serial number 187.

### Exposure variable

The exposure of interest was the FEV_1_/FVC ratio. Physiologically, younger individuals have a high ratio and must thus decrease a disproportionate amount before dropping below an FEV_1_/FVC < 70^14–16^. Similarly, if we divided participants according to FEV_1_ predicted values, the value has to decrease a disproportionate amount as already discussed by Lange et al.^14,15^. Therefore, spirometric baseline ratios were used to divide the participants into one of the following subgroups: (1) FEV_1_/FVC < 70, (2) FEV_1_/FVC: 70–75, and (3) FEV_1_/FVC > 75. Impaired lung function was defined by an FEV_1_/FVC < 70. We included all the groups in the analyses, thus we had the possibility to compare the group with FEV_1_/FVC: 70–75 with both the definitionally ill group (FEV_1_/FVC < 70) and the healthy reference group (FEV_1_/FVC > 75).

### Spirometry

Lung function was assessed using Vitalograph model R, a direct writing, 7 L, dry wedge spirometer (Vitalograph Ltd, Buckingham, U.K.)^34^, which was calibrated daily in accordance with the guidelines. The spirometer was measured FEV_1_ and FVC. At each examination, the criterion for correct procedure performance was at least three measurements differing by <5% and by evaluating the volume–time tracings. Two health professionals reviewed the quality of the spirometry independently. Only pre-bronchodilator measurements were available. We used STATA^35^ to calculate FEV_1_/FVC and the predicted value of FEV_1_ based on reference values^15^.

### Outcomes

The outcomes of this study were the number of lung-related contacts to hospitals, overall contacts to general practice, redeemed prescriptions for respiratory medicine and socioeconomic status from 1 January 1991 to 31 December 2017.

### Socioeconomic status

In Denmark, we have a unique possibility to link clinical measurements to nationwide registries and databases^29^ using the ten-digit civil registration number (CPR number)^36^ assigned to all Danish citizens. We obtained yearly socioeconomic data on income and occupation from Statistics Denmark^36^. Educational level was defined as the highest formal educational attainment categorised according to the United Nation’s Educational, Scientific and Cultural Organisation’s International Standard Classification of Education^30,37^. Educational level at baseline (1991) was categorised into the following groups: <10 years (low), 10–15 years (medium), and >15 years of education (high). Data on income level 1991–2017 were adjusted for family size using the OECD-adjusted income level. The average income is listed in Euro for every year. At baseline (1991), income was divided into tertiles to compare the groups^38^. Occupational level was available from 1991 to 2017 and was categorised into employed (including self-employed) or unemployed (including individuals on unemployment benefits or social welfare recipients). Social welfare recipients are unemployed individuals who are not members of an unemployment benefit fund or have been unemployed for >2 years. Unemployment benefits are assigned to individuals who have been unemployed for <2 years and who are members of a voluntary unemployment benefit fund. In addition, individuals were censured from the economic analyses when they started to receive retirement pension.

### Contacts to GPs

Data on contacts (daytime consultations, home visits and out-of-hour visits) to general practice were obtained from the Danish National Health Service Register^31^ from 1991 to 2017. We examined the overall annual number of consultations, including the number of consultations regarding lung symptoms (spirometry, peak flow or reversibility measurements) for each patient.

### Lung-related contacts to the hospital

Data on number of contacts to the hospital were provided by the Danish National Patient Registry^39^. Denmark used the International Classification of Diseases Version 8 (ICD-8) until 1 January 1994 and afterwards the ICD-10. Lung-related hospital contacts (outpatient and inpatient contacts) were registered using the ICD-8: 490-493, 515-518 or ICD-10: J09-18 (pneumonia and influenza), J20-22 (acute lower respiratory infections) and J40-47 (COPDs).

### Respiratory medicine

Finally, data on redeemed prescription medication was obtained from the Danish Register of Medical Product Statistics, which contains information on all redeemed prescriptions in Denmark since 1995. Data on substances and quantities are classified according to World Health Organisation within Anatomical Therapeutic Chemical (ATC) Classification system and defined daily dose methodology^40^. We included data on all respiratory medicine redeemed (ATC: R03) prescribed by a medical doctor. In Denmark, most medication, including inhalation medication, is only available on prescriptions (Supplementary Table 1). We counted the total number of redeemed prescriptions for respiratory medicine. Inclusion criteria were ≥2 prescriptions during the study period used as a proxy for treatment of lung diseases. Hence, we excluded participants with <2 prescriptions redeemed from the measurement of outcome to avoid “test” bias, *n* = 721.

All data were fully anonymised, and the performed analyses comply with the Danish regulations on registry-based research^41^.

### Statistical methods

The characteristics of the baseline participants were summarised using mean and standard deviation (SD) or proportions. Negative binomial regression models using cluster robust variance estimation were applied for the calculation of incidence rates and rate ratios and corresponding 95% CIs for comparison of hospital admissions, contacts to general practice, redeemed prescriptions for respiratory medicine and level of income. This was counted for each year during the entire period of follow-up as well as during two time periods between 1991(1995) and 2003 and between 2004 and 2017 to take the development in age of participants into account. During the 27 years of follow-up, 88 citizens died and they were censured from the analyses from the date of death. The analyses were also adjusted for sex (male, female), age (continuous) and smoking status (current, former or never) at baseline. Generalised linear models (binreg) using cluster robust variance estimations were applied for calculation of the rate ratios and corresponding 95% CI for being unemployed. All statistical analyses were performed using STATA 14^35^.

#### Additional analyses

Among the specific services provided by the GP, we investigated the activities related to lung diseases concerning peak flow/spirometry/reversibility tests (activity codes 7113, 7121, 7183). Even though we accumulated the amount for the whole period, there were too few observations to be analysed: group 1 (*n* = 287), group 2 (*n* = 437), group 3 (*n* = 2643).

We also conducted a sensitivity analysis including all individuals who had redeemed one prescription for respiratory medicine during the period (*n* = 721); this did not affect the results. Hence, we maintained the definition of respiratory medicine as two or more redeemed prescriptions for respiratory medicine during the study period.

We did not adjust for lung diseases at baseline, as none of the participants had a lung-related hospital contact in 1991 according to the Danish National Patient Registry^39^. The Register of Medical Product Statistics was first introduced in 1995 and data were thus not available at baseline.

### Ethical considerations

The study was approved by the Danish Health Data Authority, the Danish Data Protection Agency and Statistics Denmark. An informed consent form was signed by all participants before the physical health check.

### Reporting summary

Further information on research design is available in the Nature Research Reporting Summary linked to this article.

## Supplementary information

supplementary material

reporting summary

## Data Availability

The data are available from The Danish Health Data Authority but restrictions apply to the availability of these data, which were used under license for the present study, and so are not publicly available. Data are, however, available from the authors upon reasonable request and with permission of The Danish Health Data Authority and Statistics Denmark.

## References

[1] World Health Organization. in *Global surveillance, prevention and control of**Chronic Respiratory Diseases*. *A Comprehensive Approach* 1–37 (WHO, 2007).

[2] To T (2012). Global asthma prevalence in adults: findings from the cross-sectional world health survey. BMC Public Health.

[3] Soriano JB (2017). Global, regional, and national deaths, prevalence, disability-adjusted life years, and years lived with disability for chronic obstructive pulmonary disease and asthma, 1990–2015: a systematic analysis for the Global Burden of Disease Study 2015. Lancet Respir. Med..

[4] Løkke A (2014). Direct and indirect economic and health consequences of COPD in Denmark: a national register-based study: 1998–2010. BMJ Open.

[5] Borné Yan, Ashraf Wafa, Zaigham Suneela, Frantz Sophia (2019). Socioeconomic circumstances and incidence of chronic obstructive pulmonary disease (COPD) in an urban population in Sweden. COPD: Journal of Chronic Obstructive Pulmonary Disease.

[6] Çolak Y, Afzal S, Nordestgaard BG, Vestbo J, Lange P (2017). Prognosis of asymptomatic and symptomatic, undiagnosed COPD in the general population in Denmark: a prospective cohort study. Lancet Respir. Med..

[7] Guirguis-Blake JM, Senger CA, Webber EM, Mularski RA, Whitlock EP (2016). Screening for chronic obstructive pulmonary disease. JAMA.

[8] Thorn J (2012). Improved prediction of COPD in at-risk patients using lung function pre-screening in primary care: a real-life study and cost-effectiveness analysis. Prim. Care Respir. J..

[9] Frith P (2011). Simplified COPD screening: validation of the PiKo-6® in primary care. Prim. Care Respir. J..

[10] Aaron SD, Boulet LP, Reddel HK, Gershon AS (2018). Underdiagnosis and overdiagnosis of asthma. Am. J. Respir. Crit. Care Med..

[11] Martinez CH (2015). Undiagnosed obstructive lung disease in the United States associated factors and long-term mortality. Ann. Am. Thorac. Soc..

[12] Vestbo J (2013). Global strategy for the diagnosis, management, and prevention of chronic obstructive pulmonary disease: GOLD executive summary. Am. J. Respir. Crit. Care Med..

[13] Siu AL (2016). Screening for chronic obstructive pulmonary disease US preventive services task force recommendation statement. JAMA.

[14] Lange P (2015). Lung-function trajectories leading to chronic obstructive pulmonary disease. N. Engl. J. Med..

[15] Løkke A (2012). New Danish reference values for spirometry. Clin. Respir. J..

[16] Çolak Y, Løkke A, Marott JL, Lange P, Vestbo J (2013). Impact of diagnostic criteria on the prevalence of COPD. Clin. Respir. J..

[17] Oh DK (2018). Comparison of the fixed ratio and the Z-score of FEV 1 /FVC in the elderly population: a long-term mortality analysis from the third national health and nutritional examination survey. Int. J. COPD.

[18] Kotz D, Simpson CR, Viechtbauer W, van Schayck OCP, Sheikh A (2014). Development and validation of a model to predict the 10-year risk of general practitioner-recorded COPD. NPJ Prim. Care Respir. Med..

[19] Josephs L, Culliford D, Johnson M, Thomas M (2019). COPD overdiagnosis in primary care: a UK observational study of consistency of airflow obstruction. npj Prim. Care Respir. Med..

[20] Vogelmeier, C. F. et al. Global Strategy for the Diagnosis, Management, and Prevention of Chronic Obstructive Lung Disease 2017 Report: GOLD Executive Summary. *Arch. Bronconeumol*. **53**, 128–49 (2017); erratum to *Arch. Bronconeumol*. **53**, 411–412 (2017).

[21] Cerveri I (2008). Underestimation of airflow obstruction among young adults using FEV 1FVC < 70% as a fixed cut-off: a longitudinal evaluation of clinical and functional outcomes. Thorax.

[22] Bakke PS (2011). Recommendations for epidemiological studies on COPD. Eur. Respir. J..

[23] Moth G, Olesen F, Vedsted P (2012). Reasons for encounter and disease patterns in Danish primary care: changes over 16 years. Scand. J. Prim. Health Care.

[24] Thomsen M, Nordestgaard BG, Vestbo J, Lange P (2013). Characteristics and outcomes of chronic obstructive pulmonary disease in never smokers in Denmark: a prospective population study. Lancet Respir. Med..

[25] Kristensen LE (2017). Societal costs and patients’ experience of health inequities before and after diagnosis of psoriatic arthritis: a Danish cohort study. Ann. Rheum. Dis..

[26] Landfeldt E (2018). Personal income before and after diagnosis of multiple sclerosis. Value Health.

[27] Pisinger C, Jorgensen T, Toft U (2018). A multifactorial approach to explaining the stagnation in national smoking rates. Dan. Med. J..

[28] Davidsen JR (2010). Increased use of inhaled corticosteroids among young Danish adult asthmatics: an observational study. Respir. Med..

[29] Schmidt M, Pedersen L, Sørensen HT (2014). The Danish Civil Registration System as a tool in epidemiology. Eur. J. Epidemiol..

[30] Jensen VM, Rasmussen AW (2011). Danish education registers. Scand. J. Public Health.

[31] Sahl Andersen J, De Fine Olivarius N, Krasnik A (2011). The Danish National Health Service Register. Scand. J. Public Health.

[32] Haroon SM, Jordan RE, O’beirne-Elliman J, Adab P (2015). Effectiveness of case finding strategies for COPD in primary care: a systematic review and meta-analysis. npj Prim. Care Respir. Med..

[33] Lauritzen T, Leboeuf-Yde C, Lunde IM, Nielsen KD (1995). Ebeltoft project: baseline data from a five-year randomized, controlled, prospective health promotion study in a Danish population. Br. J. Gen. Pract..

[34] Vitalograph. www.vitalograph.eu (2019). Spirometer Measuring technology.

[35] Stata. Stata: data analysis and statistical software. http://www.stata.com/ (2018).

[36] Pedersen CB (2011). The Danish Civil Registration System. Scand. J. Public Health.

[37] UNESCO. International Standard Classification of Education. (2012). http://uis.unesco.org/en/topic/international-standard-classification-education-isced. Accessed 15 Mar 2020.

[38] OECD. What are the equivalence scales. Project on income distribution and poverty. http://www.oecd.org/social/inequality.htm (2011).

[39] Lynge E, Sandegaard JL, Rebolj M (2011). The Danish National Patient Register. Scand. J. Public Health.

[40] WHO Collaborating Centre for Drug Statistics Methodology. *Guidelines for ATC Classification and DDD Assignment 2013* (Norwegian Institute of Public Health, 2013).

[41] Statistics Denmark. Guidelines for transferring aggregated results from Statistics Denmark’s research services. https://www.dst.dk/en/TilSalg/Forskningsservice (2015). Accessed 3 Aug 2018.

