# Supplementary material for: Lung function in adults and future burden of obstructive lung diseases in a long-term follow-up
Source: NPJ Prim Care Respir Med. 2020 Mar 26;30:10. doi: 10.1038/s41533-020-0169-z (PMC7099055; doi:10.1038/s41533-020-0169-z)
Supplement: Supplementary file 1 — supplementary material [file 41533_2020_169_MOESM1_ESM.pdf]

Supplementary Note 1:

The Danish Ebeltoft Health Promotion Project (EHPP) was initiated in 1991. Citizens aged 30-49 years at baseline and living in the municipality of Ebeltoft were identified using their civil registration number based on their date of birth and sex. A sample of 2000 individuals was selected, randomised into three groups, and invited to attend the EHPP. A total of 1370 participated (69% participation rate) in the study; 465 participants in the control group, 449 participants in the intervention group receiving a health check only and 456 participants in the intervention group receiving both a health check and a health talk. We included 905 participants who completed a questionnaire and underwent a clinical examination at baseline. An informed consent was obtained from all participants before the clinical examination and before filling in the questionnaire. The clinical examination, including height, weight, blood pressure, blood samples, and spirometry measurement took place in a local health care center in Ebeltoft municipality and was managed by trained nurses. All the current smokers were recommended smoking cessation at the end of the health check. The second intervention group had a follow-up health talk at their own general practitioner. The self-reported questionnaire was filled in before the clinical examination and we only used data on sex, age, smoking status and respiratory symptoms within the last year.

Supplementary Table 1: Respiratory medicine only available by prescription from a medical doctor in Denmark

R03A Adrenergics, inhalants

- 1.1R03AA Alpha- and beta-adrenoreceptor agonists
- 1.2R03AB Non-selective beta-adrenoreceptor agonists
- 1.3R03AC Selective beta-2-adrenoreceptor agonists
- 1.4R03AH Combinations of adrenergics
- 1.5R03AK Adrenergics in combination with corticosteroids or other drugs, excl. anticholinergics
- 1.6R03AL Adrenergics in combinations with anticholinergics incl. triple combinations with corticosteroids

R03B Other drugs for obstructive airway diseases, inhalants

- 2.1R03BA Glucocorticoids
- 2.2R03BB Anticholinergics
- 2.3R03BC Antiallergic agents, excluding corticosteroids
- 2.4R03BX Other drugs for obstructive airway diseases, inhalants

R03C Adrenergics for systemic use

- 3.1R03CA Alpha- and beta-adrenoreceptor agonists
- 3.2R03CB Non-selective beta-adrenoreceptor agonists
- 3.3R03CC Selective beta-2-adrenoreceptor agonists
- 3.4R03CK Adrenergics and other drugs for obstructive airway diseases

R03D Other systemic drugs for obstructive airway diseases

- 4.1R03DA Xanthines
- 4.2R03DB Xanthines and adrenergics
- 4.3R03DC Leukotriene receptor antagonists
- 4.4R03DX Other systemic drugs for obstructive airway diseases

	FEV_1/FVC <70	FEV_1/FVC 70-75	FEV_1/FVC >75
n (%)	48 (27.0)	31 (17.4)	99 (55.6)
Sex (male), n (%)	16 (33.3)	16 (51.6)	36 (36.4)
Age, mean (SD)	42.9 (6.4)	40.9 (6.6)	41.7 (5.2)
Lung function			
FEV_1/FVC , mean (SD)	62.4 (6.6)	72.5 (1.5)	81.3 (4.0)
FEV_1 % predicted, mean (SD)	71.2 (15.5)	89.3 (7.4)	95.3 (10.5)
Airway symptoms within a year, n (%)			
No symptoms	7 (14.6)	15 (48.4)	63 (63.6)
Light symptoms	21 (43.8)	15 (48.4)	35 (35.4)
Server symptoms	20 (41.7)	< 5 (.)	< 5 (.)
Smoking status, n (%)			
Never smoker	8 (16.7)	< 5 (.)	28 (28.6)
Current smoker	40 (83.3)	22 (71.0)	59 (60.2)
Former smoker	0 (0.0)	6 (19.4)	11 (11.2)
Education			
0-10 years	15 (33.3)	9 (29.0)	40 (40.4)
10-15 years	24 (53.3)	17 (54.8)	43 (43.4)
15- years	6 (13.3)	5 (16.1)	16 (16.2)
Respiratory medicine, n (%)			
0-1 DDD	8(.)	11(35.5)	27(27.3)
1-2 DDD	< 5 (.)	< 5 (.)	8 (8.1)
>2 DDD	33(66.7)	6(19.4)	6 (6.1)
Lung contact, n (%)			
1-3 contacts	11(22.9)	18(58.1)	67(67.1)
4-7 contacts	10(20.8)	5(16.1)	11(11.1)
>7 contacts	27(56.3)	8(25.8)	6(6.1)

Supplementary Table 2: The table shows characteristics of the 178 individuals who had lung-related hospital contacts from 1991 to 2017.

Data are n (%), mean (SD). FEV_1= forced expiratory volume in one second. FVC= forced vital capacity. SD= standard deviation, DDD= defined daily doses

	FEV_1/FVC <70	FEV_1/FVC 70-75	FEV_1/FVC >75
n (%)	27 (14.7)	32 (17.4)	125 (67.9)
Sex (male), n (%)	13 (48.1)	20 (62.5)	48 (38.4)
Age, mean (SD)	41.5 (6.1)	40.0 (6.2)	39.9 (5.4)
Lung function			
FEV_1/FVC , mean (SD)	64.6 (4.9)	72.6 (1.3)	81.8 (4.0)
FEV_1 % pred, mean (SD)	77.0 (12.1)	88.0 (9.8)	98.7 (12.3)
Airway symptoms within a year, n (%)			
No symptoms	12 (44.4)	13 (40.6)	76 (60.8)
Light symptoms	10 (37.0)	15 (46.9)	39 (31.2)
Server symptoms	< 10 (.)	< 5 (.)	10 (8.0)
Smoking status, n (%)			
Never smoker	< 5 (.)	6 (18.8)	48 (38.4)
Current smoker	22(81.5)	21 (65.6)	63 (50.4)
Former smoker	< 5 (.)	5 (15.6)	14 (11.2)
Education, n (%)			
0-10 years	9 (34.6)	10 (32.3)	35 (28.2)
10-15 years	12 (46.2)	14 (45.2)	60 (48.4)
15- years	5 (19.2)	7 (22.6)	29 (23.4)
Respiratory medicine, n (%)			
0-1 DDD	24(88.9)	30(93.8)	117(93.6)
1-2 DDD	< 5 (.)	< 5 (.)	< 10 (.)
>2 DDD	< 5 (.)	< 5 (.)	< 5 (.)
Lung contact, n (%)			
1-3 contacts	9(21.4)	10(.)	19(46.3)
4-7 contacts	6(14.3)	< 5 (.)	9(22)
>7 contacts	27(64.3)	6(.)	13(31.7)

Supplementary Table 3: The table shows characteristics of the 184 individuals who had redeemed prescriptions for respiratory medicine from 1995 to 2017.

Data are n (%), mean (SD). FEV_1= forced expiratory volume in one second. FVC= forced vital capacity. SD= standard deviation, DDD= defined daily doses
